# Analysis of FDM and DLP 3D-Printing Technologies to Prototype Electromagnetic Devices for RFID Applications †

**DOI:** 10.3390/s21030897

**Published:** 2021-01-29

**Authors:** Riccardo Colella, Francesco Paolo Chietera, Luca Catarinucci

**Affiliations:** 1 National Research Council of Italy, 73100 Lecce, Italy; riccardo.colella@cnr.it; 2 Innovation Engineering Department, University of Salento, 73100 Lecce, Italy; francescopaolo.chietera@unisalento.it

**Keywords:** 3D-printing, DLP, FDM, T-Resonator, 3D-printed antennas, UHF, RFID

## Abstract

In this work, the application in Radiofrequency Identification (RFID) of different additive manufacturing (AM) 3D-printing technologies is discussed. In particular, the well-known Fused Deposition Modeling (FDM) technology is compared with the promising Digital Light Processing (DLP), which is based on the photopolymerization of liquid resins. Based on the research activity of the authors on this topic, a brief introduction to the fundamentals of 3D-printing in electromagnetics as well as to the different applications of both FDM and DLP in realizing Radio Frequency (RF) devices, is firstly given. Then, a comparison of the two technologies is deeply faced. Finally, after evaluated the rugosity of substrates produced with both techniques to verify the potential impact on the design of electromagnetic structures, the two techniques are both exploited for the realization of the dielectric parts of a tunable RFID tag with unconventional shape. It consists of two elements interlinked one each other. The movement between them enables tuning of the resonance frequency as well as the impedance of the antenna. Despite the differences in terms of losses, rugosity, resolution, and dielectric constant, both techniques guaranteed satisfactory values of tag sensitivity, maximum reading range, and tunability. Nevertheless, the careful analysis of the results proposed at the end of the paper suggests how the selection of one technique over the other must be taken considering the specific application constraints.

## 1. Introduction

3D printing by additive manufacturing (AM) is proving to be a promising technology to create high-detailed models wasting less time and spending fewer resources than traditional methods. In the last few years, the advent of more and more accurate and affordable 3D printers has considerably stimulated the electromagnetic community, strongly interested in the realization of microwave components and antennas which take advantage of versatility, cost-effectiveness, and ease of use of these rapid prototyping techniques.

Different 3D-printing technologies can be used to produce electromagnetic devices [1,2,3,4,5,6,7,8,9,10,11]. In Farooqui et al. and Fenn et al. [1,2], the Fused Deposition Modelling (FDM) technology has been used to build a cross-polarized (CP) microstrip fed patch antenna in Farooqui et al. [1] and a conformal antenna array in Fenn et al. [2], respectively. In the former case, the conductive parts have been obtained using copper adhesive tape, while in the latter a copper electroplating process has been carried out. In Heirons et al. and Mirmozafari et al. [3,4] a CP patch antenna and a linear array have been described as well, but this time the adopted manufacturing technology has been the Stereo Lithography (SLA) 3D-printing. In these cases, the conductive parts have been realized with inkjet printing in Heirons et al. [3] and with an electroplating process in Mirmozafari et al. [4]. In Kaddour et al. [5], a Selective Laser Sintering (SLS) process has been used to polymerize polycarbonate (PC) and ABS in other to develop a dual-polarized unidirectional wideband antenna, with metallic parts obtained with electroplating. In Gjokaj et al. [6], a Vivaldi antenna operating in the Ku band has been described as well as its manufacturing process, the so-called Polyjet 3D-printing. In this case, the metallization has been created with a first deposition by sputtering of a Titanium and Copper compound, then used as a base for an electroplating process. A different approach has been described in Jammes et al. [7], where a Jet Metal printing process has been used to produce a metal pyramidal horn antenna without the need to successively metalize anything. Similarly, in Reinhardt et al. [8] the production process of a corrugated pyramidal horn antenna has been entrusted to the Selective Laser Melting (SLM) technology. In particular, a prototype operating at 110 GHz has been realized melting Bronze powder and then plated with Gold. In Huang et al. [9] a similar technique called Direct Metal Laser Sintering (DMLS) has been used to realize a horn antenna prototype too, exploiting some Aluminum powder instead of the Bronze one. In Rojas-Nastrucci et al. [10] the Binder Jetting technology is described and used to develop a horn antenna operating in the Ka-band. Finally, in Shen et al. [11] the Digital Light Processing (DLP) has been used as production technology to manufacture some waveguide pieces easily and affordably with a very high resolution. Those pieces have then been metalized by exploiting the so-called Tollen’s reaction.

Based on an extension for a special issue of the work presented at the Splitech 2020 conference [12], this paper is focused on a comprehensive comparison between two of the most common and affordable 3D-printing techniques applied to the prototyping of electromagnetic structures. On the one hand, the FDM technique is taken into account. On the other hand, the DLP is considered. The former is based on the controlled extrusion of a fused polymeric filament which is used to build 3D objects layer by layer from bottom to top. The latter is based on the photocuring of a liquid resin which uses an LCD screen emitting UV light to harden the polymer with a resolution as high as the pixel dimension.

To better describe the pros and cons of these technologies, a brief overview of the fundamental knowledge useful for applying 3D-printing in electromagnetics is provided. In this regard, the topic of the dielectric characterization of 3D-printable materials is faced. The theoretical principle behind the T-Resonator device is described by highlighting the main steps of its possible realization taking advantage of 3D-printing. Subsequently, some considerations about the use of FDM are pointed out by focusing the attention on its limits and potentials in electromagnetics. For instance, the most important drawback of commercial printable materials when used in electromagnetic applications is their relatively low dielectric constant (between 2 and 3). Some solutions to improve this value are presented and discussed. Finally, the possibilities offered by the use of an unconventional conductive filament called Electrifi [13] which is suitable for FDM-based electromagnetic applications are discussed.

On the other hand, DLP is described and identified as one of the promising AM technologies suitable for electromagnetic applications. The differences with FDM are pointed out in terms of electromagnetic properties of the printable materials, resolution, and accuracy as well as general costs. Two prototypes of a tunable Planar Inverted F Antenna (PIFA) for Ultra High Frequency (UHF) RFID applications have been produced with both the techniques, characterized under the electromagnetic point of view and critically discussed and compared.

## 2. Electromagnetic Characterization of 3D-Printable Materials through the T-Resonator Method

In this section, a fundamental aspect of 3D-printing in electromagnetics is briefly summarized. This is the earliest problem that always arises when new materials are adopted to realize RF devices. That is the characterization of their electromagnetic properties in terms of permittivity (ε_r_) and loss-tangent (tanδ). A way to face this problem is to use the so-called T-Resonator, which is a well-known structure whose behavior is described in the literature [14,15]. It can be used to obtain a measurement instrument, as detailed in Catarinucci et al. [16], adoptable also to characterize, among the others, 3D-printed substrates. Briefly, it consists of a two-port microwave circuit composed of a microstrip line with an open-end stub resonating at odd-integer multiples of its quarter-wavelength corresponding frequency. Both the ε_r_ and tanδ of the substrate under test can be determined with a specific elaboration, once the scattering parameter S_21_ is known. For this reason, a structure capable to place a certain pressure all over the “T”, while holding still the substrate under test, has been designed and 3D-printed using common Polylactic Acid (PLA) as 3D-printable material. Screw regulators are foreseen to assure the possibility to form a sandwich structure between the metallic parts and substrate of different thicknesses. More details about the T-Resonator realization can be found in Catarinucci et al. [16].

By using the T-resonator it is possible to demonstrate how the proper tuning of the 3D-printer parameters capable of setting the “infill percentage” of a 3D-printed substrate can lead to dielectric constant customizability. Indeed, the infill percentage is a value indicating the fraction of material over air used to fill the 3D model. An object printed with a low infill percentage has a greater percentage of air inside while maintaining its usual dimensions. Indeed, by properly setting this parameter, the dielectric constant and the loss tangent of the final printed element can be controlled according to the specific application. As an example, the dependency of the PLA dielectric parameters on the infill percentage is reported in Table 1. As expected, the lower is the infill percentage, the lower are the values of dielectric constant and loss tangent of the substrate under test.

## 3. Fused Deposition Modelling in Electromagnetics

FDM is the most common 3D-printing technology and it has largely spread out in the last few years, due to the negligible cost of both printers and needed materials. It works realizing a plastic prototype extruding a melt polymeric filament through a tinny nozzle, which moves following a pattern and so drawing the proper layer shape. The final object is built layer by layer.

So far, a large literature related to the use of FDM in electromagnetics has been produced exploiting and exploring different aspects of this innovative technology. In addition to the already described works on it [1,2,16], more others could be cited. A not exhaustive list could contain Massoni et al. [17], where the authors have used the possibility to tune the infill of a model printed by FDM technology to realize a Substrate Integrated Slab Waveguide obtaining an enhanced bandwidth; Moscato et al. [18], where the same principle and the use of a special elastic material called Ninjaflex have been used to produce an unconventional antenna; Rocco et al. [19], in which the authors have described how they successfully manufactured a 3D-printed microfluidic sensor by exploiting a substrate integrated waveguide cavity; Martínez Odiaga et al. [20], where a particularly lightweight circular horn antenna for police radar application has been printed with FDM; Alkaraki et al. [21], in which authors reported the realization of a particular 3D-printed slot antenna operating in the Ka band. Eventually, in Helena et al. [22], a very accurate review on different AM technology (FDM among the others) applied in the prototyping of antennas, is proposed.

To summarize, the most interesting possibility is to prototype any kind of unconventionally-shaped electromagnetic structure, in a fraction of the time and with a lower cost than the standard manufacturing technologies.

On the other hand, one of the most important drawbacks is that, as already stated, the common commercial filaments exhibit a relatively low value of dielectric constant (between 2 and 3), and this could be not high enough in some applications where, for instance, antenna miniaturization is needed. Other problems regard the typical accuracy of an FDM printing process, which lays around 0.2 mm. In fact, although this value could be thought low enough at low frequencies, it starts to generate potential problems while increasing the frequency, especially if it affects the roughness of the conductive parts of the device.

Eventually, the possibility to realize electromagnetic devices (with both dielectric and conductive parts) only using FDM 3D-printing needs to be considered. Indeed, even if this technology allows to print only thermoplastic materials, innovative filaments with conductive properties have been recently developed and commercialized as described below.

There are two possible approaches to fulfill the need for printable devices with higher dielectric constants. The former is to take advantage of all the benefits of a shape freely designable by realizing a 3D-printed mold with standard material and then cast it with a specific compound material opportunely realized to exhibits the needed dielectric properties. The latter is to produce advanced filaments for FDM 3D-printing that overcome the limits of the standard ones. Both are described below along with the direct 3D-printing of conductive materials.

The “mold technique” is a particular method useful when flexible devices are designed. It consists in the realization of a silicone rubber compound, which is electromagnetically enhanced by adding highly dielectric powders and subsequently shaped through 3D-printed molds. One of the ceramic powders mostly used for this kind of mixture is the Barium Titanate (BaTiO_3_), already used in literature for this purpose [23] because it is a strong ferroelectric material (with a very high dielectric constant). The dielectric properties of the mixed compound can be well forecast through Lichtenecker’s equation which describes a logarithmic connection between the complex permittivity of the matrix material and that of the doping one, as can be seen in Equation (1):

(1) $$\log\varepsilon_{eff}=\log\varepsilon_{matrix}+\varphi \log\left(\varepsilon_{filler}/\varepsilon_{matrix}\right),$$

where *ɛ_eff_* is the resulting permittivity of the compound, *ɛ_matrix_* and *ɛ_filler_* are the permittivity of the matrix and the filler materials, respectively, and eventually, φ is the volume fraction of the filler in the whole composite.

**Figure 1:**
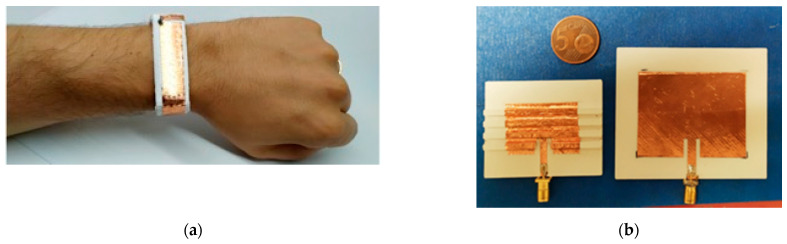
(**a**) Prototype of a ultra high frequency (UHF) radiofrequency Identification (RFID) tag for on-body application realized with the molding technique [10]. (**b**) 2.4 GHz patch antenna made with standard PLA (right) and reduced size patch antenna printed in BaTiO_3_ enhanced PLA with meandered design (left).

As an example of the results achievable with this technique, in Figure 1a the lab-made RFID tag, described in Catarinucci et al. [24], is shown. It has been realized with a compound of silicone rubber and BaTiO_3_ and has been modeled through a bracelet-shape mold.

As for the second technique based on the definition of advanced filaments with increased electromagnetic properties, such materials are realized by extruding a specific ceramic-doped 3D-printable plastic mixture. Similar to the previous case, the main idea consists again in increasing the dielectric constant of a base material by adding highly dielectric powders. This technique has been previously used to realize an ABS filament doped with BaTiO_3_ as reported in Yingwei et al. and Castles et al. [25,26]. Conversely, the authors of the present work experienced the PLA as a matrix instead of ABS, because of its greater ease of print.

Even in this case, Lichtenecker’s equation can proficiently forecast the final dielectric constant, which is revealed to be, at the same percentage of doping agent, slightly lower than the one obtained by using silicone rubber. This is obviously due to the lower starting value of the dielectric constant of the PLA (~2.5 for PLA vs. ~3.2 for silicone).

The outcome is a printable filament that achieves ε_r_ ≃ 4.6 and tanδ ≃ 0.015 for a doping percentage of 17.5% of the total volume. This is a remarkable result considering that the dielectric constant of a standard PLA has been almost doubled maintaining acceptable values for the losses. As an example of application, in Figure 1b comparison between a 2.4 GHz patch antenna realized with common PLA and one printed using the BaTiO_3_ enhanced PLA joint with a particular meandered design for the patch radiating element, is shown. It is clear how the improved dielectric constant and the clever design guarantees a considerable size reduction of the antenna.

In addition to the above-discussed techniques to improve the material permittivity, one of the most interesting possibilities enabled by 3D-printing is to extrude the conductive parts to obtain novel fully 3D-printed electromagnetic devices, without the need for other techniques to produce the metallic parts. Obviously, the main type of material which is usable by the desktop 3D-printers is a polymer that is naturally not a conductor. Nevertheless, in the last few years, some unconventional hybrid materials, composed of polymer and nanoparticles of conductors, have been developed and commercialized. One of the most promising is Electrifi, which is produced by Multi3D [13]. With a declared conductivity of 1.6 × 10^4^ S/m, and a measured one ranging from 1.2 × 10^3^ S/m to 8.3 × 10^3^ S/m (it depends on the printing settings and the measurement direction), it revealed to be a good enough conductor for realizing fully 3D-printed RF devices.

**Figure 2:**
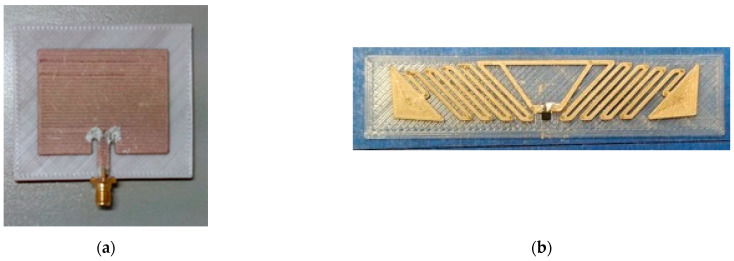
(**a**) Fully 3D-printed 2.4 GHz patch antenna made of PLA (substrate) and Electrifi (antenna) [13]. (**b**) Fully 3D-printed UHF RFID tag made of PLA (substrate) and Electrifi (antenna) [28].

As an example of the achievable results, one of the first prototypes of a fully 3D-printed patch antenna, operating at around 2.4 GHz and printed in Electrifi and PLA, is shown in Figure 2a. This structure is better discussed in Colella et al. [27], while the prototype of similarly realized UHF RFID antenna is shown in Figure 2b and deeply compared with other similar-layout tags in Colella et al. [28].

## 4. Digital Light Processing in Electromagnetics

As stated, the most common technology for 3D-printing prototyping and, hence, for 3D-printing in electromagnetics, is the FDM one. However, it is not the only affordable one. In fact, especially in the last few years, the resin-based (also known as vat polymerization) 3D-printing technology has spread out also in the consumer market. Specifically, the cost of these types of printers has dropped down with the advent of Digital Light Processing (DLP). It briefly consists of the photopolymerization of the resin using a UV light emitted by a cost-effective high-resolution display. Hence, the substitution of the most common and expansive light source (which, for example, is a laser in SLA 3D-printers or a projector in another type of DLP machines) as well as the consequent simplification of the required mechanic, led to a consistent drop of the costs maintaining the quality guaranteed by the resin-based 3D-printing techniques. DLP, indeed, allows one to achieve a level of accuracy in the model realization even 10 times higher than the FDM one. This characteristic is particularly appreciable when a complex geometry is required. Moreover, a lower-dimensional tolerance is an added value in microwave device realization, where even little discrepancies between the simulated and the realized device could lead to not tolerable errors (typically when frequencies are over 10 GHz and surface roughness of the device affects the conductivity).

**Figure 3:**
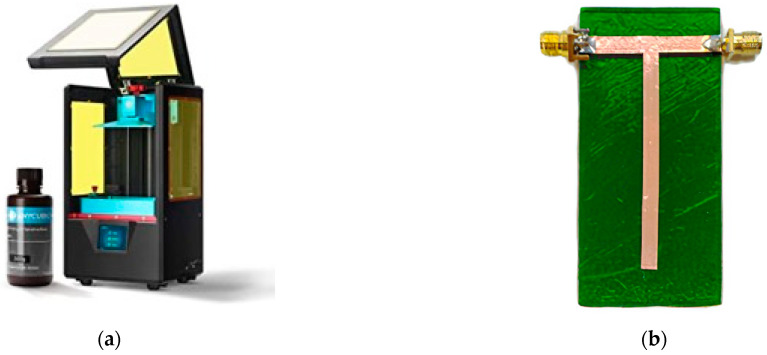
(**a**) Digital Light Processing (DLP) 3D-printer Anycubic Photon-S with its own 405 nm resin. (**b**) Resin-made T-Resonator printed with a DLP 3D printer.

As for the printable materials for FDM technology, the authors have made a preliminary study to characterize the dielectric properties of one common commercial DLP resin (Anycubic Green 405 nm) [23]. An Anycubic Photon-S DLP 3D printer (see Figure 3a) has been used to produce a 40 × 80 × 1.6 mm^3^ substrate, using an Anycubic 405 nm resin. Then, a copper adhesive tape has been shaped into a properly dimensioned “T” (considering the substrate height the microstrip width has been set to be 4.2 mm) through a cutting plotter and subsequently applied to the previously printed substrate, so to obtain the resonator shown in Figure 3b.

The 52 mm length of the stub allows one to measure the dielectric properties of the polymer at a frequency around 800 MHz, near the working band of the UHF RFID technology, on which the further described application is focused. Specifically, values of ε_r_ = 3.11 and tanδ = 0.033 have been obtained.

## 5. Analysis of FDM and DLP Technologies in Electromagnetics

Analyzing the main properties of the 3D-printing technologies described in the previous sections, it is possible to compare them in terms of the electromagnetic properties of the printable materials, resolution of the final printed parts and, general costs. After this theoretical comparison, a more practical one is carried out by confronting two UHF RFID tunable antennas realized with the same conceptual design but using the two different rapid prototyping methods.

### 5.1. Electromagnetic Properties of the Printable Materials

Comparing the results obtained by the dielectric characterization, through the T-Resonator method, of both common DLP and FDM materials it is clear how the resin exhibits a higher value for dielectric constant, but also for loss tangent. However, even if the losses of this specific resin resulted relatively high, they are nevertheless compatible with a real application and the use of DLP remains appealing since it allows to realize geometries that are unprintable otherwise.

The possibility of making improved versions of printable materials by adding, for example, ceramic powder to the polymer matrix is a viable path for both techniques. Specifically, in the case of FDM, it is possible to realize a printable filament as stated in Section 3, while for the DLP technique a mix of resin and powder can be used instead of simple resin to photopolymerize the model [29]. In the latter case, different printing settings must be used to successfully compensate for the minor penetration distance of the light in a compound doped with an opaque powder. A good starting point could be to enhance of 50% the curing time of both the firsts model layers (which have to attach robustly to the moving bed of the printer) as well as the following ones.

### 5.2. Resolution and Accuracy

To successfully understand the difference in accuracy between the two techniques, it is useful to understand which are the elements that affect it. For example, in FDM the two main elements that determine the printing accuracy are the nozzle diameter (ranging from 0.1 to 1 mm) and the layer height (approximately ranging from 0.08 to 0.64 mm). The former affects the surface roughness of the material (along the *XY* plane), the latter affects the roughness along the *z*-axis. Moreover, while the latter can be easily taken low, paying accuracy with printing-time, the former is a parameter not so easily tunable. In fact, a lot of materials cannot be printed through a too-small nozzle, and for this reason, the highest accuracy for an FDM 3D-printer is considered to be around 0.2 mm.

Conversely, the DLP has a single moving part, which is the *z*-axis stepper that moves the printing bed. A typical value for the accuracy of this stepper is 0.0125 mm. On the other hand, the accuracy on the *XY* plane is given by the size of the LCD pixels, which for the DLP printer used by authors is 0.047 mm.

Finally, as for the roughness of the printed surface, it has to be considered that it is the same as the Fluorinated Ethylene Propylene (FEP) film that is used as vat bottom surface of the DLP 3D-printer. This allows DLP printers to produce models with very smooth surfaces if compared with that realized with an FDM process (a more detailed analysis is performed in the “Results” Section).

### 5.3. General Costs

The strength of modern AM technologies like FDM and DLP is the very low costs of both printers and materials. For example, the FDM 3D-printer used to realize the prototypes proposed in this work is purchasable for about $200, while for less than $400, it is possible to buy a DLP 3D-printer. As for the consumables, the cost of a spool of filament for FDM ranges from $20 to $200, depending on the material properties. Even for a bottle of resin for DLP a similar amount of money is needed. This makes these AM techniques appealing for rapid prototyping at a fraction of the cost and time of standard production methods.

## 6. UHF RFID Tunable Antennas: Design and Realization

**Figure 4:**
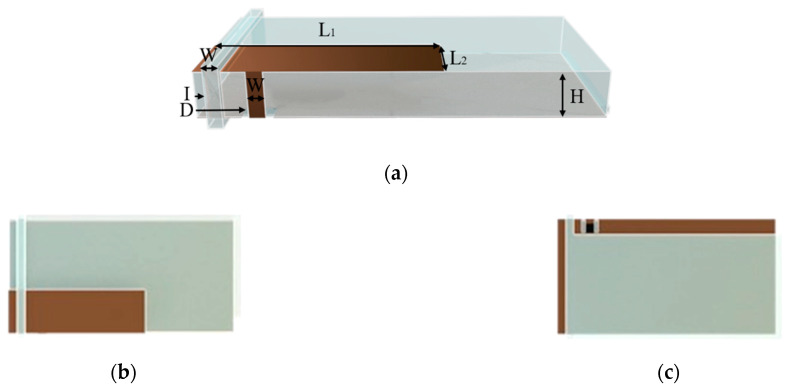
Rendered image of the designed Planar Inverted F Antenna (PIFA) antenna. (**a**) Prospective view. (**b**) Top view. (**c**) Bottom view.

A particular type of PIFA layout has been designed and simulated in CST Microwave Studio. A self-explaining rendered image can be observed in Figure 4. Briefly, the device is composed of 2 elements: the body, where the radiating element, as well as the feeding line and the background plane, are placed, and the sliding ring, which wraps around the body and has copper attached on its inner side. The body can float inside the ring, while the copper on it acts as shorting wall for the PIFA. The movement allows to vary the distance between the shorting wall and the feeding line of the antenna, thus determining a tuning procedure that can proficiently adapt the antenna parameters depending on the needed application. It is worth highlighting that a similar layout is enabled by the 3D-printing technology.

It is worth highlighting that the prototype printed with the DLP printer has been realized using a common commercial resin. Conversely, a particular built-in-lab filament composed of PLA doped with a 17.5% volume fraction of BaTiO_3_ has been used for the prototype realized with FDM technology. The filament has been produced mixing and extruding at the same time the Ingeo 4043D (NatureWorks, Minnetonka, MN, USA) PLA pellet with a BaTiO_3_-325 mesh powder pure at 99%, through a twin-screw extruder. The doping percentage has been selected to have a good compromise between dielectric constant increment and ease of printing. Indeed, as the amount of BaTiO_3_ increases, the brittleness increases as well, and the ease of flow of the material is reduced. This mix guarantees a dielectric constant of 4.8 at around 900 MHz and a loss tangent of 0.015 for the substrate of the antenna, helping to reduce antenna size if compared with a standard PLA substrate, as well as making the device more platform tolerant.

Conversely, the prototype made with the commercial resin, exhibiting a lower value of dielectric constant (3.11), had a larger size (see Table 2). Moreover, without loss of generality, for the DLP prototype, an additional brace has been added to the ring part so to better ensure the stiffness of the prototype.

**Figure 5:**
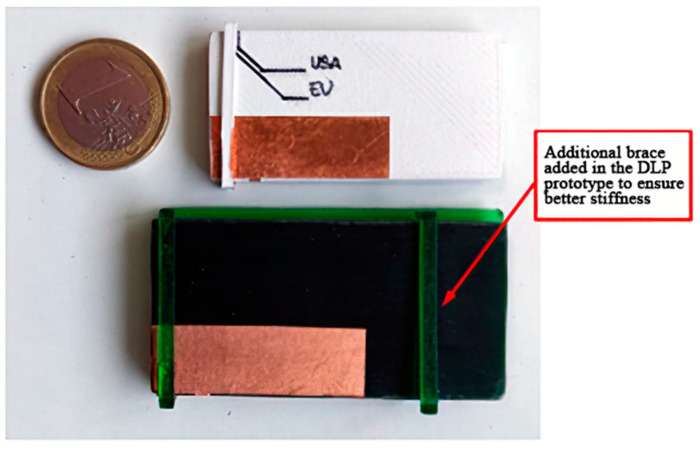
Final 3D-printed prototypes of the UHF RFID PIFA inspired antenna.

As for the metallic parts of the antennas, they have been realized with a tiny copper adhesive tape-shaped using a Graphtec ce6000-40 (Graphtech Corporation, Tokyo, Japan) cutting plotter [30]. In Figure 5 the two realized prototypes are shown.

Eventually, as for the internal chip (IC), The simulation phase has been aimed at optimizing the parameters of the antennas to match the impedance of the Impinj Monza R6 chip (Impinj, Seattle, WA, USA) [31].

## 7. Results

**Figure 6:**
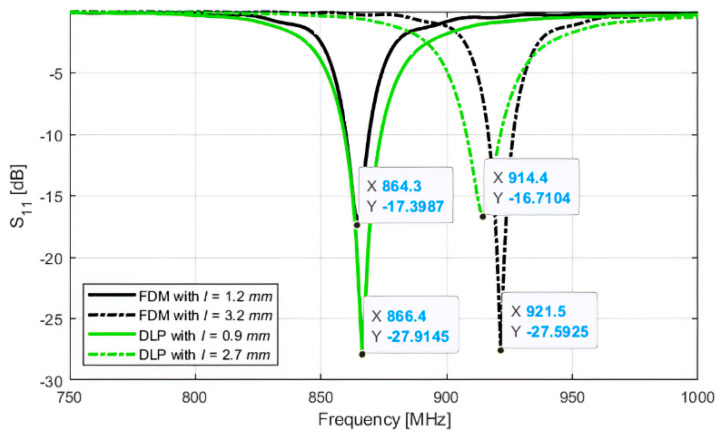
Comparison between Fused Deposition Modelling (FDM) and DLP roughness profiles.

First of all, to take into account all possible differences between the two realization techniques, a roughness measure has been made as shown in Figure 6, where two roughness profiles of respectively an FDM and a DLP 3D-printed sample are shown. They have been measured with a Veeco Dektak 150 (Veeco, Plainview, NY, USA) equipped with a 12.5 nm stylus and the compared profiles have been chosen to be the ones with the highest values (so to consider the worst case). As can be easily seen, the DLP sample has a roughness of barely one order of magnitude lower than the FDM sample. Specifically, the RMS values for both the profiles are respectively 2.15 μm for the DLP and 12.82 μm for the FDM. Nevertheless, these values are barely negligible if a UHF RFID application is developed, because they are hugely smaller than the wavelength at working frequencies. For this reason, simulations have been performed without taking the roughness of the material into account.

**Figure 7:**
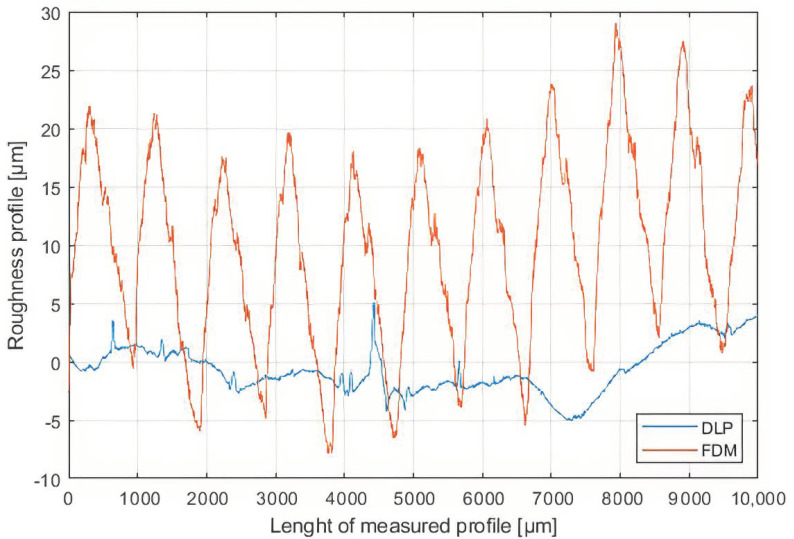
Comparison between the simulated S_11_ curves for the antennas in both the working configurations.

For both the antennas, the S_11_ curve for the two different configurations of the tag, corresponding to different positions of the wrapping ring (referred to the different values of parameter I in Table 2), have been simulated as shown in Figure 7. As can be seen, each antenna exhibits two points of good matching, respectively for working in the ETSI or FCC bands. These simulations have been confirmed by the sensitivity measurements that have been used to have a snapshot of the two tags’ performance with varying frequency.

**Figure 8:**
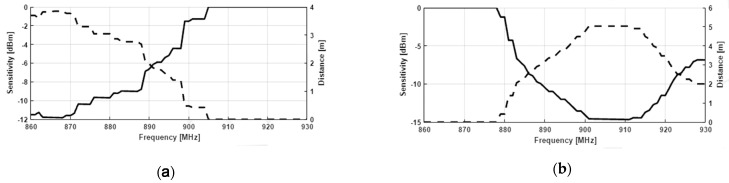
Sensitivity and maximum reading distance for FDM 3D-printed prototype, respectively for ETSI (**a**) and FCC band (**b**).

**Figure 9:**
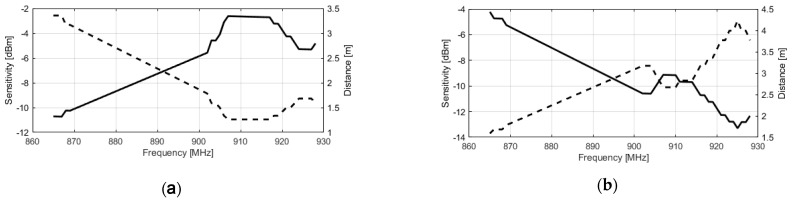
Sensitivity and maximum reading distance for DLP 3D-printed prototype, respectively for ETSI (**a**) and FCC band (**b**).

The measurements have been made using the built-in-lab instrument described in Colella et al. [32] and the results in terms of sensitivity as well as maximum reading distance curves are shown in Figure 8 and Figure 9. Analyzing the curves, it is clear how the change in the position of the ring part of the antennas, undoubtedly determines a tune of the antenna impedance which allows it to operate correctly at the selected frequency. Moreover, a slightly better performance is obtained by the antenna printed with the FDM method, despite its smaller size. This is probably due to the lower value of loss-tangent of the used filament (~0.015) that is barely half the one of the commercial resin (Anycubic Green 405 nm) used for the DLP prototype (~0.033).

## 8. Comments and Future Directions

The sensitivity and maximum reading distance described in the previous section allow one to state that both the examined AM technologies can be suitable for realizing electromagnetic devices with similar performance, at least at the UHF RFID frequencies. Nevertheless, the two technologies have not to be considered interchangeable. There are indeed many differences between them that must be considered when choosing one over the other to realize a specific RF application. For example, when higher frequency applications are needed the difference in resolution could play a major role, especially if it impacts the roughness of the conductive parts of the device. Moreover, considerations about losses must be also considered. The commercial resins seem to show higher losses if compared with commercial FDM filaments; however, an improvement of their electromagnetic properties by using for instance high dielectric constant and low losses ceramic powders as filler deserves to be further investigated.

On the other hand, FDM takes advantage of the larger diffusion and of the longer research effort of the scientific community to improve it from the electromagnetic point of view. This determines a larger variety of materials and techniques available for it which, for example, makes it possible to print flexible or elastic structures (using materials as the so-called Ninjaflex [33]), or fully 3D-printed conductive parts (using for instance the Electrifi [13]).

There is no doubt that, despite its current limits, DLP technology has the potential to contribute to the realization of 3D-printed electromagnetic devices, overcoming the resolution limits of the more widespread FDM technology. However, dedicated research is needed to make new materials, techniques, and procedures suitable for electromagnetic projects available for DLP technology.

## 9. Conclusions

In this paper, a comparison between the two most used and cheap 3D-printing techniques is carried out highlighting the most useful aspects from the electromagnetic point of view. Other than a recap of the fundamentals of 3D-printing in electromagnetics and a theoretical discussion about the differences between FDM and DLP, a more practical comparison has been proposed. Specifically, it consists of the analysis of the performance of two prototypes of the same tunable PIFA antenna, designed for UHF RFID applications and realized with both the rapid prototyping methods under test.

In the end, the results have been commented on and used to discuss the potential of the research activity in the framework of 3D-printing of electromagnetics, as well as its future development.

## Figures and Tables

**Table 1:** Dielectric PLA parameter with varying the infill percentage.

Infill %	ε_r_	tanδ
20	1.503	0.0031
30	1.578	0.0034
40	1.646	0.0035
50	1.8	0.0043
60	1.942	0.0048
70	2.13	0.0051
80	2.25	0.0054
90	2.371	0.0062
100	2.541	0.0071

**Table 2:** Design parameters of the two different antenna prototypes (size in [mm]).

Parameters	DLP	FDM
L1	36.6	30.5
L2	11.6	10.5
W	2.4	2
I	0.9 ^1^/2.7 ^2^	1.2 ^1^/3.2 ^2^
D	7.8	6.5
H	6	6

^1^ For the configuration operating in the European Telecommunications Standards Institute (ETSI) band. ^2^ For the configuration operating in the Federal Communications Commission (FCC) band

## Data Availability

The data presented in this study are available on request from the corresponding author.
